# Propofol-Related Infusion Syndrome: A Clinical Review

**DOI:** 10.7759/cureus.30383

**Published:** 2022-10-17

**Authors:** Aayushi Singh, Ashish P Anjankar

**Affiliations:** 1 Anesthesiology, Jawaharlal Nehru Medical College, Datta Meghe Institute of Medical Sciences, Wardha, IND; 2 Biochemistry, Jawaharlal Nehru Medical College, Datta Meghe Institute of Medical Sciences, Wardha, IND

**Keywords:** myoglobin, creatine kinase, ecg, cellular hypoxia, beta-oxidation, mitochondrial respiratory chain, steroids, catecholamines, critical illness, propofol infusion syndrome

## Abstract

Propofol-related infusion syndrome (PRIS) is a lethal condition characterized by multiple organ system failures. It can occur due to prolonged administration of propofol (an anesthetic) in mechanically intubated patients. The main presenting features of this condition include cardiovascular dysfunction with particular emphasis on impairment of cardiovascular contractility, metabolic acidosis, lactic acidosis, rhabdomyolysis, hyperkalaemia, lipidaemia, hepatomegaly, acute renal failure, and eventually mortality in most cases. The significant risk factors that predispose one to PRIS are: critical illnesses, increased serum catecholamines, steroid therapy, obesity, young age (significantly below three years), depleted carbohydrate stores in the body, increased serum lipids, and most importantly, heavy or extended dosage of propofol. The primary pathophysiology behind PRIS is the disruption of the mitochondrial respiratory chain that causes inhibition of adenosine triphosphate (ATP) synthesis and cellular hypoxia. Further, excess lipolysis of adipose tissue occurs, especially in critically ill patients where the energy source is lipid breakdown instead of carbohydrates. This process generates excess free fatty acids (FFAs) that cannot undergo adequate beta-oxidation. These FFAs contribute to the clinical pathology of PRIS. It requires prompt management as it is a fatal condition. The clinicians must observe the patient's electrocardiogram (ECG), serum creatine kinase, lipase, amylase, lactate, liver enzymes, and myoglobin levels in urine, under propofol sedation. Doctors should immediately stop propofol infusion upon noticing any abnormality in these parameters. The other essentials of management of various manifestations of PRIS will be discussed in this article, along with a detailed explanation of the condition, its risk factors, diagnosis, pathophysiology, and presenting features. This article aims to make clinicians more aware of the occurrence of this syndrome so that better ways to manage and treat this condition can be formulated in the future.

## Introduction and background

Propofol

Propofol (commonly sold under the brand name Diprivan®) is the most frequently used hypnotic intravenous anaesthetic drug that aids in maintaining procedural sedation and hypnosis in general anaesthesia during surgeries. Propofol's pharmacokinetic characteristics make it a desirable alternative for tranquilising intubated patients in intensive care units who are mechanically ventilated. Further, its quick onset of action and brief half-life enables faster awakening after discontinuing the infusion. In addition to all these uses of propofol, it is significant to mention that we can readily adjust the dosage of propofol in critically ill patients to maintain an optimal degree of sedation [1]. Even though it finds its use in multiple surgeries, its utmost significance comes from providing anaesthesia to patients who undergo ambulatory and neurosurgical procedures. These procedures require an instant revival of consciousness and motor functions [2,3]. One can give propofol in the form of an infusion or bolus or as a joint combination of both. The preparation of propofol involves a lipid emulsion that confers an appearance of a whitish milky substance, giving it a popular name in the field of anaesthesia, i.e., 'milk of amnesia' [4-7].

*Mechanism* *of* *Action*

**Figure 1:**
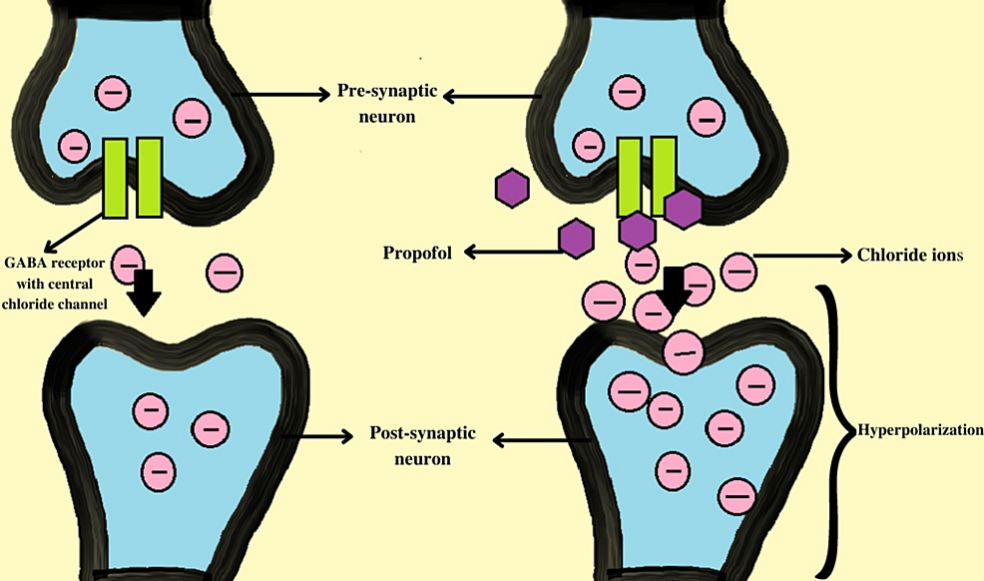
Mechanism of action of propofol Propofol acts by binding to the GABA receptors and increasing the duration of their action. This leads to prolonged opening of the central chloride channels that causes hyperpolarization of post-synaptic neurons, thereby making it difficult for an action potential to fire. (The figure shown above has been made by the authors of this article).

Propofol works by enhancing the neuro-inhibitory activity of GABA, i.e., gamma-aminobutyric acid, a global inhibitory neurotransmitter of the central nervous system. Propofol specifically acts on the GABA-A receptors that are known to have several subunits (α1, β1, γ, δ, ε, and ρ) which are phylogenetically related and form a pentamer consisting of chloride channels in the centre. Propofol binds to these receptors, prolongs the duration of action of GABA, and the period of opening of these chloride channels causes hyperpolarization of the following postsynaptic neuronal membrane leading to their subsequent inhibition. This process results in hypnosis and sedation, as expected by the drug [4,8-10]. The mechanism has been depicted in Figure 1.

*Adverse* *Effects* *of* *Propofol*

Propofol which is so popularly used for anaesthesia comes with its own risks. The most common side effect associated with infusion of propofol is pain at the site of injection and the most significant adverse reaction is airway obstruction and apnea which makes this drug highly unsafe in unprofessional or untrained hands. Other side effects of this drug include the incidence of bradycardia, hypotension, central nervous system excitation which can even precipitate a seizure, alterations in serum lipids, and infections of the bloodstream. In this article, we’ll be talking about PRIS which is, as the name suggests, a condition characterized by a group of symptoms that occur due to overdosage or prolonged sedation with propofol. It is characterized by circulatory collapse and severe metabolic acidosis. It was first seen in paediatric population and later on in the adults as well [11].

What is propofol-related infusion syndrome?

Propofol-related infusion syndrome (PRIS) is a potentially fatal condition caused due to prolonged exposure to propofol for sedation and it is primarily marked by severe cardiorespiratory depression along with a high level of metabolic acidosis. This adverse bodily reaction to propofol was first reported in a 3-year-old Danish girl in 1990. Since then, having been presented mainly in the paediatric population and causing serious neurodevelopmental damage to children below three years of age, PRIS has also made its way into the adolescent, adult, and old-aged populations [12]. As the number of paediatric cases rose, the US, the UK, and several other regions ceased using propofol for long-term sedation among children. The first adult mortality by PRIS occurred in 1998 in an asthmatic patient whose condition worsened because of long-term propofol sedation for mechanical pulmonary ventilation [13]. The chief systems of the body affected by PRIS are cardiovascular, metabolic, hepatic, renal, and musculoskeletal. The main presenting signs of PRIS include high anion gap metabolic acidosis (due to lactic acidosis and renal failure), rhabdomyolysis (in skeletal and cardiac muscles), cardiac dysfunctions, hypertriglyceridemia, lipaemia, hyperkalaemia and hepatomegaly [12,14,15]. In this article, we'll focus in-depth on the risk factors, pathophysiology, clinical presentation, investigations, management, and prevention of PRIS.

## Review

Risk factors for the development of propofol-related infusion syndrome

Poor oxygen saturation, sepsis, traumatic brain injury, ongoing critical illness, young age, elevated catecholamines, inborn errors of metabolism, usage of corticosteroids, an imbalance between lipid and carbohydrate stores of the body, and heavy propofol dose are the foremost risk factors for the emergence of PRIS [1]. These risk factors can be listed and explained as follows:

Heavy or Long-Term Dosage of Propofol

One must not give propofol for more than 48 hours or at a dose greater than 4 mg/kg/hour (or 67 mcg/kg/minute) according to evidence provided by case reports and studies. Dosage for durations longer than this has proven to be fatal and is not recommended [12]. It is better to switch to alternative drugs for sedation instead of propofol if required.

Elevated Catecholamines

Vasopressor therapy and the occurrence of PRIS are related. The elevated levels of endogenous catecholamines observed in patients with cerebrovascular injuries, those with an abnormally increased circulatory volume due to sepsis, or those undergoing a systemic inflammatory response, can lead to the acceleration of the clearance of propofol. This can also happen when we give inotropic or vasopressor infusions in more significant amounts. Propofol's therapeutic index may be diminished by the higher clearance rate, thereby necessitating an increase in the drug's prescribed dosage, this can lead to PRIS. Further, acidosis brought on by PRIS may decrease vasomotor tone, increasing the need for vasopressors [16].

Younger Age Group

Children below three years of age experienced severe neurodevelopmental damage, leading to behavioural changes and abnormalities as they grew up. Also, death due to PRIS was found to be more prevalent in the population <18 years of age group as children, infants and neonates are much more sensitive to intravenous anaesthetics than the adult age group [17].

Ongoing Critical Illness

Catecholamines and glucocorticoids spike up due to the neuroendocrine stress response to severe and critical illnesses (acute neurological injury, status epilepticus, pancreatitis, sepsis, etc.), which forms one of the major predisposing factors for PRIS. These hormones are responsible for the regulation of the enzyme activity of lipase, which facilitates the conversion of triglycerides into glycerol and free fatty acids (FFAs). Additionally, the energy source manufactured in the body shifts from carbohydrate to lipid in critically ill patients, which further results in an increase in FFAs. This is of significance because FFAs play a crucial role in the pathophysiology of PRIS [1,12,16].

Excess Lipids or Dearth of Carbohydrates

Patients can develop a lipid excess state because of parenteral feeding, propofol infusion (as propofol is itself a lipid emulsion), or a combination of both. If dextrose infusion is not used to regulate excess lipolysis and maintain a balance, then there'll be a collection of FFAs in the body and a depletion of the glycogen stores. This will give rise to the pathophysiological mechanism of PRIS which will be explained in the next section of this article [16,18].

Usage of Corticosteroids

The usage of corticosteroids is another risk factor [19]. The mechanism behind steroids leading to PRIS lies in the activation of the ubiquitin-proteasome pathway that causes muscle rupture because of myofilament disturbance [20]. There is also proof that steroids alter gene transcription, which lowers mitochondrial energy output by altering the mitochondrial pathways and functions. Hence, corticosteroids serve as an essential risk factor for the development of PRIS [13,21,22].

Individuals Suffering From Inborn Errors of Metabolism

Patients suffering from inborn errors of metabolism [17], especially mitochondrial diseases, come under a great risk for developing PRIS upon long-term exposure to propofol. This is because PRIS causes defects in the mitochondrial respiratory chain. Hence, a child with an already compromised mitochondrial function must be closely monitored before propofol sedation [23]. 

**Figure 2:**
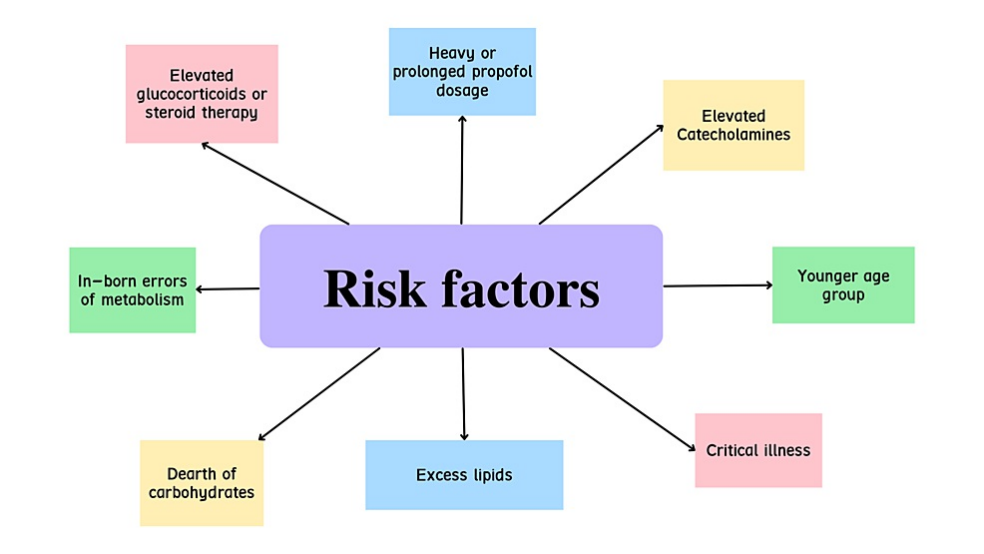
Risk factors for the development of PRIS PRIS: Propofol-Related Infusion Syndrome (The figure shown above has been made by the authors of this article).

A flowchart of all the risk factors has been depicted in (Figure 2).

Pathophysiology of propofol-related infusion syndrome

The mechanism responsible for PRIS remains controversial [24]. Propofol hinders the uptake and usage of FFAs and mitochondrial activity at molecular and cellular levels. The poor balance between energy requirement and consumption is a fundamental pathogenetic process that can cause cardiac and perivascular muscle damage [25]. One of the prevailing theories posits that propofol impairs the electron transport chain or the respiratory chain function, which in turn leads to the collapse of the body's metabolic activities [26]. According to various theories, propofol decouples oxidative phosphorylation and obstructs the flow of electrons in the electron transport chain running through the inner-mitochondrial membrane [27]. What exactly happens is that the electrons from the reduced coenzymes nicotinamide adenine dinucleotide (NADH) and succinate, which are generated from the citric acid cycle, reach the electron transport chain (ETC) via complexes I and II respectively, during oxidative phosphorylation. Electrons are subsequently sent to complex III, cytochrome C, and complex IV via coenzyme Q. This electron movement causes proton translocation and accumulation in the intermembranous region of the mitochondria, resulting in an electromechanical gradient that promotes ATP production by ATP synthase. Propofol has been found to block complex I, complex IV, cytochrome c, and the acylcarnitine transferase in the mitochondria and it also serves as an uncoupling agent in the oxidative phosphorylation process [23]. Hence, the faulty operation of the electron transport chain lowers the mitochondrial capacity for energy production and might result in an imbalance between demand and energy utilization. It also leads to the production of several long, medium, and short-chain fatty acid metabolites. It should also be noted that when the body faces immense metabolic insults and stress, FFAs obtained from lipolysis of adipose tissues (as explained above, catecholamine and glucocorticoid-mediated) become the chief energy providers for musculoskeletal and cardiovascular cells. These FFAs undergo beta oxidation inside the mitochondria to generate acetyl-coenzyme A, which is needed to sustain one of the most crucial ATP-generating processes of the body, i.e., the Krebs Cycle or the Citric Acid Cycle. This cycle leads to the generation of several free electrons which enter the electron transport chain and as discussed above, at high dosages, propofol hinders the pathways of these electrons and leads to lowered transmembrane potential, inhibition of ATP production, cellular hypoxia and metabolic acidosis [24-26].

Another process or theory by which the mitochondrial electron transport chain is hindered states that long-term administration of propofol causes a rise in serum malonylcarnitine, responsible for the inhibition of carnitine palmitoyl transferase-1 (CPT-1) which is a mitochondrial transport protein. This creates a problem because unlike the medium- and short-chain fatty acids, the long-chain fatty acids cannot move by diffusion and require CPT-1 to get transported into the matrix. Hence, the impairment of their movement leads to long-chain fatty acid accumulation in the mitochondrial matrix causing insult to the respiratory chain and subsequently resulting in cellular hypoxia and metabolic acidosis [13]. Also, the long-chain acyl-carnitine esters too require CPT-1 for transport and henceforth are unable to traverse into the muscular tissue cells, which leads to muscle necrosis and associated rhabdomyolysis of PRIS [28-30]. The respiratory chain is impeded by excess medium and short-chain fatty acids that diffuse into the mitochondria coming from propofol (propofol contains soya that is rich in fatty acids) [31-33]. Consequently, there occurs impaired fatty acid oxidation causing a concentration of hazardous fatty acid intermediates, which exacerbates acidosis when combined with cellular hypoxia [32,33]. The high levels of FFAs also precipitate ventricular arrhythmia as seen in PRIS [34,35].

**Figure 3:**
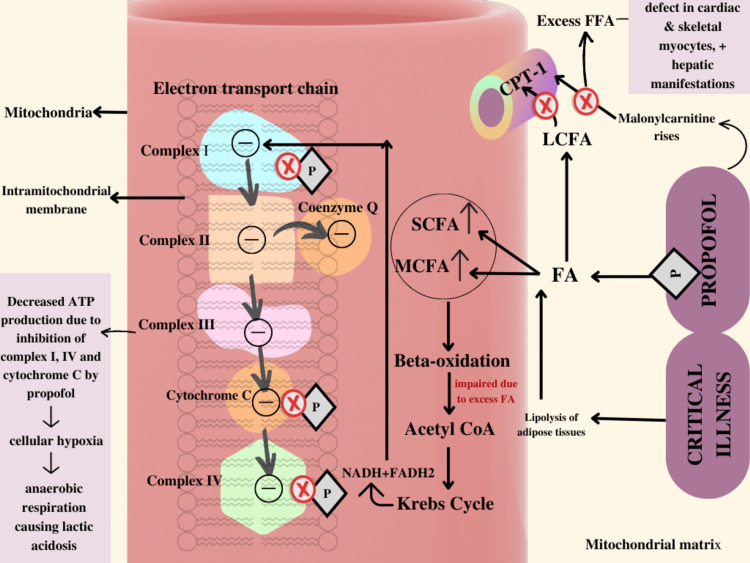
Pathophysiological mechanism of PRIS FA - Fatty Acid, SCFA - Short Chain Fatty Acid, MCFA - Medium Chain Fatty Acid, LCFA - Long Chain Fatty Acid, FFA - Free Fatty Acid, CPT-1 - Carnitine Palmitoyl Transferase-1. (The figure shown above has been made by the authors of this article).

Together, these activities affect cellular oxygen and substrate supply, disrupt fatty acid oxidation, hinder ATP generation, and even cause cell death and necrosis [1]. The mechanisms are shown in Figure 3.

Clinical presentation of propofol-related infusion syndrome

PRIS presents a wide range of features with multi-organ involvement. The most significantly involved systems are the metabolic and cardiovascular systems in adults and children [13]. Other organ systems involved include hepatic, renal, and musculoskeletal systems. The metabolic manifestations are a result of tissue-level hypoxia. The high FFAs and triglycerides lead to a 'fat overload syndrome' wherein the hydrolysis isn't able to balance out the triglycerides in circulation, as a result of which these triacylglycerols get collected in the blood and are absorbed by the reticuloendothelial system leading to hepatomegaly, splenomegaly, jaundice, coagulation disorders, etc. Rhabdomyolysis is a result of defective beta-oxidation of fatty acids as previously discussed in the pathophysiological mechanism of PRIS [36]. Multiorgan failure and cardiac failure are more than often late and deadly manifestations of PRIS [37].

Cardiovascular Manifestations

The cardiovascular system suffers from depleted ATP stores and high FFAs, which directly attack cardiomyocytic functions [36]. Due to its antagonistic effects on beta-adrenoreceptors and calcium channels, propofol diminishes sympathetic tone, which results in bradycardia and impaired myocardial contractility that can even lead to asystole [34,38]. Further, the ventricular arrhythmias associated with PRIS can be understood by the pro-arrhythmic effects of excess serum FFAs [26,35]. The several cardiovascular manifestations are as follows: right bundle branch block, hypotension, brugada-like syndrome ECG presentation (elevated ST segment and widening of QRS complex), ventricular tachycardia, ventricular arrhythmia, supraventricular tachycardia, atrial fibrillation, cardiogenic shock and asystole [12,15].

Metabolic Manifestations

These include metabolic acidosis, lactic acidosis, hyperkalaemia, hypertriglyceridemia, and hyperthermia. Metabolic acidosis further worsens hyperkalaemia because of the rise in transcellular shift [12].

Hepatic Manifestations

The hepatic changes may be associated with hepatic congestion, which can often be seen secondary to cardiac failure in PRIS. Also, the high levels of lipid deposition from propofol and free circulating fatty acids due to excessive lipolysis (hyperlipidaemia) are further responsible for the enlargement of the liver (hepatomegaly) and other hepatic features that are seen in PRIS [26,39,40]. The various hepatic manifestations are hepatomegaly; steatosis; elevated liver enzymes alanine aminotransferase (ALT), aspartate aminotransferase (AST), and gamma-glutamyl transferase (GGT); hyperlipidaemia; hypertriglyceridemia; and liver failure. Further, it is noticed that hypoperfusion, hypoxia, sepsis, hypermetabolic states, and vasopressor therapy involved in this syndrome also cause impairment of liver function and exaggerate hyperlipidaemia [26,41].

Renal Manifestations

The renal disorders of PRIS include acute kidney injury and renal failure [12,17].

Musculoskeletal Manifestations

PRIS causes extreme lysis of myocytes in the entire musculoskeletal system. On histological examination, one can find highly necrosed myocytes with unorganized myofibrils, sarcomeres, degenerated nuclei, absence of striations, and swelling. These are typical signs of rhabdomyolysis, leading to renal failure and myoglobinuria. Further, it is also suggested that decreased oxygen supply, leading to anaerobic metabolism, is a primary cause of death of myocytes that eventually causes raised serum creatinine and rhabdomyolysis associated with PRIS [25,26].

Investigations for diagnosis of propofol-related infusion syndrome

The investigations necessary for the diagnosis of PRIS are shown in Table 1.

**Table 1:** Investigations required for the diagnosis of PRIS PRIS: Propofol-Related Infusion Syndrome Table references have been taken from - [26,42]

BEDSIDE	LABORATORY
ECG - Brugada-like pattern showing coved ST elevation in V1-V3 right precordial leads and widening of QRS Complex.	Lipid profile – elevated (lipemic serum)
Arterial blood gas analysis - decreased pH, unexplained lactic acidosis, hyperkalemia.	Liver enzymes (AST, ALT, GGT) – elevated
Blood pressure - Low (Hypotensive)	Creatinine kinase – elevated (due to rhabdomyolysis)
Respiratory rate – higher than normal	Serum lactate – elevated
Heart rate – less than 60 beats per minute (acute bradycardia that can progress to asystole)	Myoglobin in urine – elevated
Body temperature – higher than 38°C (febrile)	Serum lipase and amylase - elevated

Management and treatment of propofol-related infusion syndrome

It is very important for clinicians to look for early signs of PRIS and better try to prevent it as most of the cases of PRIS end in mortality. Clinicians must not administer propofol for more than 48 hours and a dosage of 4mg/kg/hour at a stretch. They must also look out for all the risk factors that have been mentioned above in this article.

The first step in managing PRIS is to immediately recognise and discontinue propofol infusion and replace it with other sedating agents like alfentanil, midazolam, etc. [14]. Metabolic and lactic acidosis can be managed by the administration of sodium bicarbonate, haemodialysis, and hemofiltration. The latter two also help in flushing out the excess lipids in the blood. The cardiac manifestations can be managed with cardiac pacing, inotropes, and vasopressors that’ll help in improving the contractility of cardiomyocytes and counteracting the low blood pressure, and managing cardiogenic shock. Oxygenation and circulatory support can also be provided via ECMO i.e., extracorporeal membrane oxygenation [1,12,14,26]. ECMO is similar to cardiopulmonary bypass machines used in open heart surgeries. In ECMO, blood is pumped out of the vascular system into the extracorporeal membrane unit. Here, oxygen saturation of the haemoglobin is improved and carbon dioxide is drained out. The rejuvenated blood is then returned back to circulation. This greatly helps in managing metabolic acidosis and overall circulation to the organs [43]. For managing the severe hyperkalaemia associated with PRIS, the administration of calcium, insulin, beta-2 agonists, and potassium-binding resins can be taken into account as well [12].

Prevention of propofol-related infusion syndrome

Since PRIS is a seemingly rare phenomenon, hence, spreading awareness among doctors about its occurrence and being alert are the best ways to prevent it from happening [17]. Propofol dosages should be kept as low as safely practicable and within the therapeutic margin. Other medications should be taken into account for critically ill patients, in intensive care units, who need prolonged sedation. Pharmacists, critical care physicians, anaesthesiologists, and trainees should be aware of the likelihood of this uncommon but dangerous event due to the significant mortality associated with PRIS. When a long duration of propofol infusion is necessary for sedating severely ill patients, it s advised to closely monitor the significant markers of PRIS in circulation, such as arterial blood gas levels, lactic acid levels, electrolyte levels, and signs of cardiac dysfunction manifested by cardiac arrhythmias and hypotension. This is done to ensure there is no toxicity [37]. Apart from this, maintaining a balanced load of carbohydrates can help to counter the increased FFA levels and thus, help in preventing PRIS [17]. Further, given the similarity in the pathophysiology of the two conditions, propofol should be avoided in patients with known or suspected mitochondrial disorders [16]. We can also reduce the risk of occurrence of PRIS by using a solution of propofol which is more concentrated (around 60 mg/ml), this will help in reducing the lipid load [26,44].

## Conclusions

In conclusion, even though propofol is a popular choice for sedating mechanically intubated patients, it should be used judiciously by clinicians as it is a dose and duration-sensitive drug and can lead to a fatal condition known as propofol-related infusion syndrome if used for a very long duration or given in improper dosages. The anaesthesiologists must maintain vigilance for the risk factors of PRIS such as total doses of propofol, younger age group, elevated catecholamines, usage of corticosteroids, ongoing critical illnesses, inborn errors of metabolism, and excess lipids or lack of carbohydrate stores in patients under mechanical ventilation who are undergoing propofol sedation. The patient should be closely monitored for the development of signs and symptoms of PRIS which include metabolic acidosis with lactic acidosis, severe bradycardia, ventricular arrhythmia, asystole, cardiogenic shock, rhabdomyolysis, hepatomegaly, acute kidney injury, renal failure, hypertriglyceridemia, hyperkalaemia, hyperlipidaemia, elevated liver enzymes, increased serum creatinine kinase, serum lipase, amylase, lactate, myoglobinuria and Brugada-like ST elevation in ECG to name the important ones. The pathophysiology of PRIS lies in the defective mitochondrial respiratory chain and faulty beta-oxidation of excess fatty acids that lead to a decrease in ATP synthesis causing cellular hypoxia, metabolic acidosis, and other signs associated with PRIS. If a patient collapses into cardiorespiratory and metabolic distress due to PRIS, early management and treatment must be implemented. Propofol cessation, switching to an alternative drug such as vasopressors, inotropes, hemodialysis, and ECMO remain the mainstay of treatment for PRIS. This article has attempted to provide a detailed account of PRIS pertaining to its risk factors, clinical features, pathophysiological mechanism, diagnosis, treatment, and prevention. It serves to improve the knowledge and clinical instincts of the clinicians making them more alert and aware of this syndrome and its management.
